# A Coffin-Siris syndrome–associated mutation modeled in *Caenorhabditis elegans* affects multiple developmental processes

**DOI:** 10.1093/g3journal/jkaf194

**Published:** 2025-08-20

**Authors:** Marissa Baccas, Jun Liu

**Affiliations:** Department of Molecular Biology and Genetics, Cornell University, Ithaca, NY 14853, United States; Department of Molecular Biology and Genetics, Cornell University, Ithaca, NY 14853, United States

**Keywords:** Coffin-Siris syndrome, soxC, SEM-2, HLH-8, twist, *C. elegans*, postembryonic mesoderm, m lineage, SWI/SNF, BAF

## Abstract

Coffin-Siris syndrome (CSS) is a rare human genetic disorder that is characterized by developmental delay, fifth digit abnormalities, and craniofacial defects. Heterozygous mutations in 2 SoxC proteins, SOX4 and SOX11, are associated with this disorder. *Caenorhabditis elegans* has a single SoxC protein, SEM-2, which is essential for development. In this study, we use *C. elegans* as a model system to explore the molecular effects of one CSS-associated SOX11 mutation, Y116C, on SoxC protein function in vivo. The equivalent amino acid of SOX11 Y116 is SEM-2 Y160, a residue in the C-terminal tail of the highly conserved DNA-binding domain. Homozygous, but not heterozygous, *sem-2[Y160C]* animals exhibit a high rate of embryonic and larval lethality, egg-laying defects, reduced brood size, bivulval phenotype and a low penetrance of hermaphrodite tail abnormalities. Additionally, *sem-2[Y160C]* animals have reduced expression of *hlh-8/Twist,* whose human counterparts, when mutated, are known to be associated with craniofacial disorders. All the phenotypes observed in *sem-2[Y160C]* animals resemble SEM-2 loss-of-function phenotypes, suggesting that *SOX11[Y116C]* is a loss-of-function, recessive mutation that likely causes defects due to haploinsufficiency. Our work suggests that using *C. elegans* as a model system to analyze the molecular effects of point mutations associated with craniofacial defects has the potential for unraveling the underlying mechanisms.

## Introduction

Coffin-Siris syndrome (CSS) is a rare human genetic disorder with patients displaying an array of phenotypes. CSS is characterized by coarse craniofacial features, intellectual disability, hypoplastic fifth fingernails or toenails, hypoplastic or absent fifth distal phalanges, hypotonia, hirsutism, feeding difficulties and frequent infections (Fleck et al. 2001; Schrier et al. 2012). Recent advances in molecular genetics have also attributed other deformities to CSS, such as short stature, external ear deformity, ambiguous genitalia and anorectal malformations (Xia et al. 2023; Alharbi et al. 2024; Veazey et al. 2024; Wu et al. 2024). Some CSS patients display more severe developmental defects than others or a different combination of defects (Schrier et al. 2012; Schrier Vergano 2024; Apicella et al. 2025). Overall, the presentation of CSS in patients varies widely, presumably due to the different effects of the causative mutations and the different genetic backgrounds (Kosho et al. 2014).

CSS is inherited in an autosomal dominant pattern, but it is most often caused by *de novo* mutations in components of the BAF, also known as the SWI/SNF, chromatin remodeling complex, including ARID1A, ARID1B, ARID2, DPF2, SMARCA2, SMARCA4, SMARCB1, SMARCC2, SMARCE1, as well as in PHF6, which functions cooperatively with BAF (Vergano and Deardorff 2014; Mittal et al. 2024). Mutations in 2 SoxC proteins, SOX4 and SOX11, have also been found to be associated with CSS (Vergano and Deardorff 2014; Schock and LaBonne 2020). Most CSS-associated *SOX4* or *SOX11* gene variants are either point mutations or deletions, consistent with decreased activities of SOX4 or SOX11 (Tsurusaki et al. 2014a; Hempel et al. 2016; Zawerton et al. 2019). However, the functional consequences of these specific CSS-associated *SOX4* or *SOX11* variants have not been tested as heterozygous mutations under physiological conditions in vivo. Additionally, ∼40% of individuals with CSS do not have a pathogenic variant in one of the identified genes (Vergano and Deardorff 2014). This lack of diagnosis highlights the need for expansion of the gene regulatory network underlying CSS, which could potentially lead to the identification of novel biomarkers for molecular diagnosis and/or the design of therapeutics.

*Caenorhabditis elegans* is a valuable organism for modeling rare genetic disorders because of its invariant cell lineage, transparent body, short life cycle, well-annotated genome, hermaphrodite reproduction and rich set of genetic manipulation techniques (Baldridge et al. 2021; Kropp et al. 2021; Mew et al. 2022). Importantly, *C. elegans* has homologs for 60% to 80% of human genes (Sonnhammer and Durbin 1997; Kuwabara and O'Neil 2001). Further, of the 2,466 human disease genes in the ortholog detection algorithm, OrthoDisease, 533 orthologs have been identified in *C. elegans* (O'Brien et al. 2004; Kaletta and Hengartner 2006). Lastly, since the *C. elegans* genome is relatively small, containing ∼100 Mb and ∼20,000 protein-coding genes, it often contains a single ortholog for a group of paralogous human proteins (Shaye and Greenwald 2011), which minimizes the hindrance of redundancy when studying the functions of proteins.

In this study, we use *C. elegans* as a model system to examine the effects of a heterozygous CSS-associated mutation, SOX11[Y116C], on SoxC function. SoxC proteins are highly conserved transcription factors, and *C. elegans* contains a single SoxC protein, SEM-2, which is encoded by *sem-2* (Tian et al. 2011). SEM-2 is essential for embryonic and postembryonic development (Tian et al. 2011; Baccas et al. 2025). Previous studies have shown that one copy of *sem-2* is sufficient for an overall wild-type phenotype (Tian et al. 2011). This makes it convenient for us to use *C. elegans* to determine whether heterozygous CSS-associated mutations in humans cause a phenotype due to a dominant gain-of-function effect, a dominant-negative effect, or haploinsufficiency. Our results suggest that the dominant effect of *SOX11[Y116C]* in humans is most likely due to haploinsufficiency. We further showed that this mutation in *C. elegans*, when homozygous, affects the expression of *hlh-8*, the ortholog of the human Twist genes, which are known to be critical for craniofacial development. The relationship between SoxC proteins and Twist expands the gene regulatory network underlying CSS and supports the use of *C. elegans* as a model system for analyzing the molecular effects of point mutations that are associated with craniofacial disorders.

## Materials and methods

### *C. elegans* strains and transgenic lines

*Caenorhabditis elegans* strains were maintained at 20 °C. Animals carrying the *hlh-8(jjIs3900[hlh-8p::nls::mCherry])* reporter were placed at 25 °C for 2 to 3 h prior to imaging and scoring. This temporary temperature shift did not affect the mutant phenotype but improved our ability to detect very faint, if there was any, *hlh-8p::nls::mCherry* signal in *sem-2[P158S]* and *sem-2[Y160C]* mutant animals. Strains used in this study are listed in Table 1.

**Table 1.** *Caenorhabditis elegans* strains used in this study.

Strain ID	Genotype
LW5915	*sem-2(jj321) I*
LW7170	*hT2[qIs48] (I;III)/+ I*
LW7171	*hT2[qIs48] (I;III)/+ I; jjIs3900 [hlh-8p::nls::mCherry::lacZ + myo-2::mCherry] IV*
LW7172	*hT2[qIs48] (I;III)/sem-2(jj471[Y116C]) I*
LW7173	*hT2[qIs48] (I;III)/sem-2(jj473[Y116C]) I*
LW7174	*hT2[qIs48] (I;III)/sem-2(jj473[Y116C]) I; jjIs3900 [hlh-8p::nls::mCherry::lacZ + myo-2::mCherry] IV*
LW1926	*hT2[qIs48] (I;III)/sem-2(ok2422) I isolate #1*
LW1928	*hT2[qIs48] (I;III)/sem-2(ok2422) I isolate #2*
LW6502	*hT2[qIs48] (I;III)/sem-2(ok2422) I; jjIs3900 [hlh-8p::nls::mCherry::lacZ + myo-2::mCherry] IV*

### Sequence alignment and structural modeling

Sequences of SoxC proteins were obtained from UniProt (https://www.uniprot.org/). Sequence alignment was made using Clustal Omega Multiple Sequence Alignment (https://www.ebi.ac.uk/jdispatcher/msa/clustalo). The structure of the mouse SOX4 DNA-binding domain-DNA complex was obtained using PDB code 3U2B (Jauch et al. 2012).

## CRISPR

CRISPR was performed by injecting the ribonuclear protein (RNP) complex, a mixture containing Cas9 protein, tracr RNA, 2 CRISPR RNAs, a single-stranded DNA oligo repair template (all ordered from IDT), and pRF4(*rol-6(d)*) (Mello and Fire 1995), which was used as a co-injection marker. Injections were performed according to the protocol in (Beacham et al. 2022). Injected animals were singled onto NGM plates seeded with OP50 bacteria. Plates that gave the most roller progeny were selected for screening by PCR using primers JKL-1910, JKL-1911 and MDB-118. JKL-1910 and JKL-1911 are flanking primers located outside of the repair template sequence, while MDB-118 only anneals to the mutated sequence but not the WT sequence. Thus, PCR using the 3 primers will generate 2 fragments (instead of one fragment for WT) when using template DNA from animals that carry the mutation. Final genotyping was carried out using primers JKL-1910 and JKL-1911 followed by Sanger sequencing to confirm homozygosity of the *sem-2[Y160C]* mutation. Oligos used for CRISPR are listed in Table 2.

**Table 2.** Oligos used in this study.

Oligo ID	Sequence
CRISPR RNAs used for generating *sem-2(jj471[Y160C])* and *sem-2(jj473[Y160C])*	
sgRNA-MDB-7	TTCTTCTTTGGCTTCTTGCG
sgRNA-MDB-8	CGTGGCTTGTATTTGTAGTC
Repair oligo for generating *sem-2(jj471[Y160C])* and *sem-2(jj473[Y160C])*	
MDB-106*	GTAACCGGATTTTTCGAATAATTGTATTGTAATTTTAAATTTTTTCAGGAATATCCAGAtTgCAAgTAtAAaCCACGtAAaAAGCCgAAaAAGAAcCCAGATGGAACACTTCAGCAGCCAGCTCAACCCCAAGCTC
Oligos used for genotyping *sem-2(jj471[Y160C])* and *sem-2(jj473[Y160C])*	
JKL-1910	GTCAGTGTAGGAGGTAGGTG
JKL-1911	GGCATCTTTTGTGCATGACTC
MDB-118	tTgCAAgTAtAAaCCACGt

^*^Lower case letters indicate changes introduced to mutate Y160 to C160, and to Wobble bases of adjacent amino acids to prevent Cas9-gRNA re-cutting of the repair template.

### Brood size and lethality assays

To score for brood size, L4 animals were singled onto NGM plates seeded with OP50 and allowed to give progeny. For normal egg-laying animals, the parents were transferred to a new plate every 24 h for 4 d, and the total number of progeny on plates was tallied. For egg-laying defective animals, the total number of eggs within the parent and the newly hatched L1s on each plate was tallied after 24 to 48 h. To score embryonic and larval lethality, the total number of surviving L4-adult progeny on each plate was counted after 2 to 3 d. The total number of surviving L4-adults was compared with the total brood size for each singled parent to calculate the lethality for each strain.

### Microscopy

Epifluorescence and differential interference contrast microscopy was performed using a Leica DMRA2 compound microscope equipped with a Hamamatsu Orca-ER camera and the iVision software (Biovision Technology). Image analyses were performed using Fiji/ImageJ. For comparison of fluorescence intensities in different genetic backgrounds, images were collected at the same magnification and exposure.

### Statistical analysis

Statistical significance was calculated using unpaired 2-tailed Student's *t*-tests and ANOVA with a Tukey's HSD (Honestly Significant Difference) test using Prism10 (https://www.graphpad.com).

## Results

The *C. elegans* equivalent mutation for the CSS-associated *SOX11[Y116C]* mutation is *sem-2[Y160C].*

**Fig. 1.**
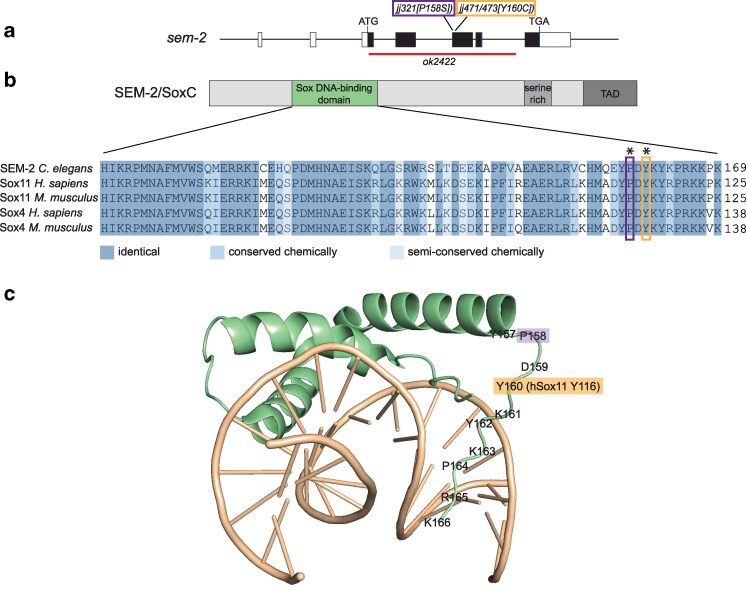
The CSS-associated SOX11[Y116C] mutation affects a highly conserved residue in the Sox DNA-binding domain. a) Schematics showing the exon-intron structure of the *sem-2* gene and the locations of the molecular lesions of various *sem-2* alleles. Black boxes indicate the coding region, and white boxes indicate the 5' or 3' untranslated regions. b) The structure of SEM-2/SoxC proteins, including the Sox DNA-binding domain (green), the serine-rich region (light gray), and the transactivation domain (TAD, dark gray), and a sequence alignment of the SoxC DNA-binding domains in SEM-2 and mammalian SOX4 and SOX11. SEM-2 Y160/SOX11 Y116 is indicated with an orange box, and SEM-2 P158/SOX11 P114 is indicated with a purple box. Both amino acids are also marked with asterisks. c) Structural model of the SEM-2 DNA-binding domain (green) in complex with DNA (tan), based on the structure of the Mouse SOX4 DNA-binding domain-DNA complex (PDB code 3U2B, Jauch et al. 2012). SEM-2 P158 is highlighted in purple, while SEM-2 Y160 is highlighted in orange, with the equivalent human SOX11 Y116 indicated in parentheses.

SoxC proteins have a conserved DNA-binding domain, a serine-rich region, and a transactivation domain (TAD) (Fig. 1a). We have previously shown that a single amino acid change in the DNA-binding domain of the *C. elegans* SoxC protein SEM-2, P158S, significantly compromises SEM-2 function (Baccas et al. 2025). Protein sequence alignment shows that SEM-2 P158 is equivalent to human SOX11 P114 (Fig. 1b). We were intrigued that a heterozygous mutation in human *SOX11*, Y116C, is associated with CSS (Tsurusaki et al. 2014a). SOX11 Y116 is a highly conserved residue in the DNA-binding domain of not only all SoxC proteins but also all Sox domain-containing proteins (Remenyi et al. 2003; Prokop et al. 2012), and its equivalent residue in *C. elegans*  SEM-2 is Y160 (Fig. 1b). Based on structural homology modeling with the mouse SOX4 DNA-binding domain (Jauch et al. 2012), SOX11 Y116 or SEM-2 Y160 is located specifically in the flexible C-terminal tail region of the DNA-binding domain (Fig. 1c). Because of our previous research on the *sem-2[P158S]* mutation (Baccas et al. 2025), we decided to use the CRISPR/Cas9 system to generate mutant animals carrying the Y160C mutation in *sem-2* and determine the consequences. We generated 2 independent alleles, *sem-2(jj471[Y160C])* and *sem-2(jj473[Y160C]*).

### Homozygous *sem-2[Y160C]* mutants are egg-laying defective and display highly penetrant embryonic and larval lethality

**Fig. 2.**
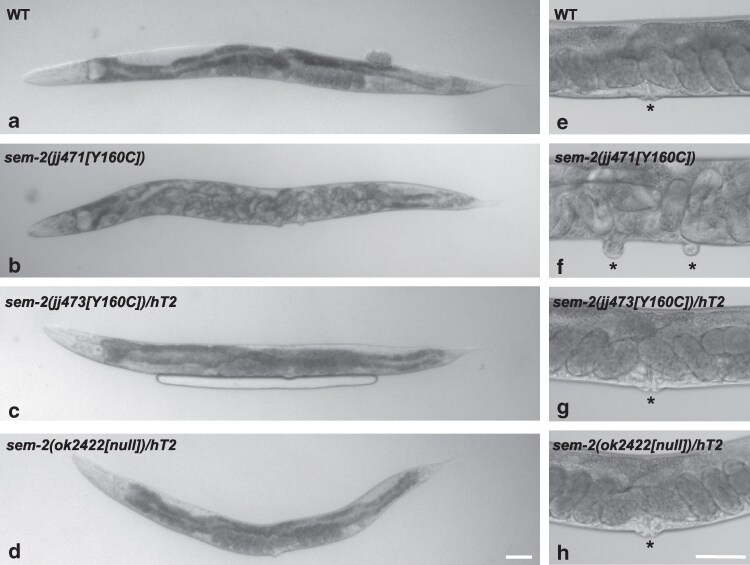
*sem-2[Y160C]* mutants are egg-laying defective with a bivulval phenotype. a–h) DIC images of gravid adults (a–d) and their vulva regions (e–h) of wild-type (a, e), *sem-2(jj471[Y160C])* (b, f), *sem-2(jj473[Y160C])/hT2[qIs48]* (c, g) and *sem-2(ok2422[null])/hT2[qIs48]* (d, h) animals. Images show the egg-laying defective (Egl) and bivulval phenotypes of *sem-2(jj473[Y160C])* animals (in b, f). Asterisks mark the vulvae. Scale bars, 50μm.

Homozygous *sem-2(jj471[Y160C])* and *sem-2(jj473[Y160C]*) animals are 100% egg-laying defective (Egl); they do not lay eggs (Table 3; Fig. 2b). Instead, their progeny hatch inside the parent, creating the bag-of-worms phenotype. Homozygous *sem-2(jj471[Y160C])* and *sem-2(jj473[Y160C]*) animals also have a reduced brood size, likely due to the Egl phenotype (Table 3). In addition, homozygous *sem-2(jj471[Y160C])* and *sem-2(jj473[Y160C]*) animals display ∼65.7% embryonic and larval lethality, compared with wild-type (WT) animals that display no lethality (Table 3).

**Table 3.** The *sem-2[Y160C]* mutation results in embryonic and larval lethal, egg-laying defective (egl), and bi-vulval phenotypes.

Genotype	Brood size (n)	% Embryonic and larval lethality^a^ (n)	% Egl (n)	Number of vulvae			
				0 (n)	1 (n)	2 (n)	3 (n)
WT (*+/+*)	229 ± 24 (10)	0 (2582)^b^	0 (>100)	0	50	0	0
*sem-2(jj471[Y160C])*	37 ± 10 (19)****	68.1 ± 11.1 (712)****	100 (>100)	2	33	34	3
*sem-2(jj473[Y160C])*	38 ± 11 (16)****	63.3 ± 9.3 (615)****	100 (>100)	0	34	24	2
*sem-2(jj321[P158S])*	37.3 ± 5.2 (10)^b^ ****	30.31 ± 9 (373)^b^ ****	100 (>100)	0^b^	335^b^	25^b^	0^b^
*sem-2(ok2422[null])*	N/A	100^c^	N/A	N/A			
WT (*hT2/+)*	248 ± 83 (7)	N/A^d^	0 (79)	0	50	0	0
*sem-2(jj471[Y160C])/hT2*	299 ± 64 (8)^ns^	N/A^d^	3.37 (89)	0	93	3	1
*sem-2(jj473[Y160C])/hT2*	321 ± 59 (7)^ns^	N/A^d^	2.14 (81)	0	47	0	0
*sem-2(ok2422[null])/hT2*	234 ± 54 (6)^ns^	N/A^d^	6.25 (80)	0	106	0	0

^a^Lethality given is the total embryonic and larval lethality in the progeny of the parental genotype that is listed. The average brood size and average percentage of lethality ± standard deviation are presented in this table.

^b^Data from Baccas et al. (2025).

^c^Data from Tian et al. (2011).

^d^Embryonic lethality was not scored due to the high degree of aneuploidy caused by the hT2 balancer chromosome (Edgley et al. 2006).

*hT2* refers to *hT2[bli-4(e937) let-?(q782) qIs48(Pmyo-2::gfp; Ppes-10::gfp; Pges-1::gfp)],* which is a reciprocal translocation between chromosome I and chromosome III (Edgley et al. 2006).

n, number of worms scored. For brood size, n reflects the number of parents that were singled and scored for their brood sizes. Details on scoring the brood size and embryonic lethality are described in Materials and methods.

^****^ *P* < 0.0001 when compared with WT based on unpaired 2-tailed Student's *t*-test. ns, not significant. Homozygous *sem-2* mutant animals were compared to WT (*+/+*) animals, while heterozygous *sem-2/hT2* animals were compared with WT (*hT2/+)* animals.

N/A, not applicable.

A possible contribution to the Egl phenotype of *sem-2[Y160C]* mutants is their abnormal vulval phenotype. Previous studies have revealed a role of *sem-2* in vulval development, with *sem-2(RNAi)* animals and *sem-2[P158S]* mutants having vulval defects (Tian et al. 2011; Baccas et al. 2025). Indeed, 38.7% of *sem-2(jj471[Y160C])* animals are bivulval, with 4.8% having 3 vulvae, while 40% of *sem-2(jj473[Y160C])* animals are bivulval, with 3.3% having 3 vulvae (Table 3; Fig. 2f).

### *sem-2[Y160C]* mutants have reduced expression of *hlh-8/Twist*

**Fig. 3.**
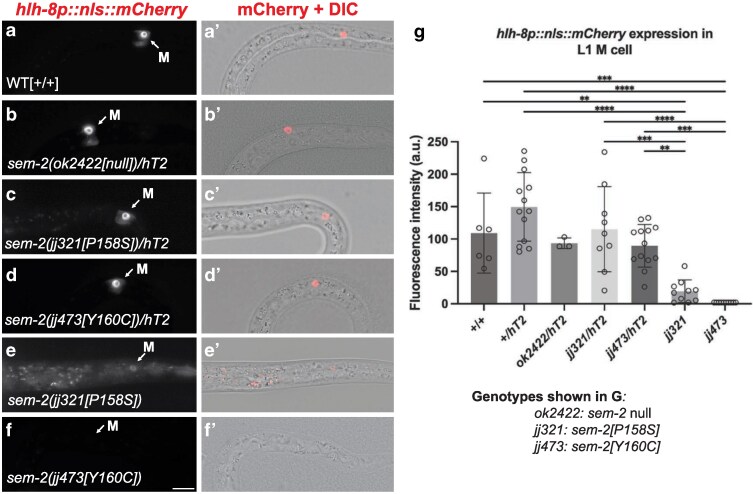
*sem-2[Y160C]* mutants have reduced expression of *hlh-8/Twist.* a–f’) Fluorescence (a–f) or merged fluorescence/DIC (a’–f’) images showing the expression of *jjIs3900[hlh-8p::nls::mCherry]* in the M cell of wild-type (a, a’), *sem-2(ok2422[null])/hT2[qIs48]* (b, b’), *sem-2(jj321[P158S])/hT2[qIs48]* (c, c’), *sem-2(jj473[Y160C])/hT2[qIs48]* (d, d’), *sem-2(jj321[P158S])* (e, e’) and *sem-2(jj473[Y160C])* (f, f’) animals. Scale bar, 10μm. g) Quantification of *jjIs3900[hlh-8p::nls::mCherry]* expression in the M cell of various *sem-2* mutants. Each dot represents a nucleus. Data are normalized to WT. *jjIs3900[hlh-8p::nls::mCherry]* was undetectable in *sem-2(jj473[Y160C])* mutants. Scoring for *sem-2(ok2422[null])* can be found in Baccas et al. (2025). Statistical significance was calculated by performing ANOVA with a Tukey's HSD. ****P < 0.0001, ***P < 0.001, **P < 0.01.

Another underlying reason for the Egl phenotype of *sem-2[Y160C]* mutants is abnormal development of the postembryonic mesoderm lineage, the M lineage. The M lineage gives rise to all the vulval and uterine muscles required for egg-laying in *C. elegans* hermaphrodites (Sulston and Horvitz 1977). We have previously shown that SEM-2 regulates the expression of the *C. elegans* Twist ortholog *hlh-8* in the M lineage (Baccas et al. 2025). HLH-8 is known to be important for the proper patterning of the M lineage and the proper differentiation of the egg-laying muscles, such that *hlh-8(0)* null mutants are Egl (Corsi et al. 2000). We therefore examined *hlh-8* expression in the M mesoblast cell in *sem-2[Y160C]* mutants using a transgenic *hlh-8* transcriptional reporter *jjIs3900[hlh-8p::nls::mCherry]* (Shen et al. 2017). We found that there is significantly reduced expression of *hlh-8p::nls::mCherry* in *sem-2(jj473[Y160C])* mutants compared with wild-type animals (Fig. 3A, A’, F, F’, G). These data demonstrate that residue Y160 plays an important role in the ability of SEM-2 to activate the expression of the *hlh-8* transcriptional reporter.

### *sem-2[Y160C]* mutants display a low penetrance of constipation and hermaphrodite tail defects

**Fig. 4.**
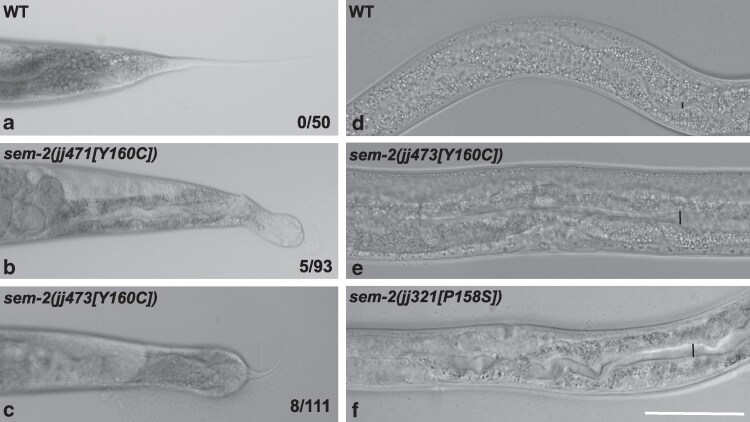
*sem-2[Y160C]* hermaphrodites exhibit a low penetrance of abnormal tail and constipation phenotypes. DIC images showing the tail (a–c) and intestinal lumen (d–f) of wild-type (a, d), *sem-2(jj471[Y160C])* (b), *sem-2(jj473[Y160C])* (c, e), and *sem-2(jj321[P158S])* (f) animals. Black bars in d–f indicate the diameter of the intestinal lumen. Scale bar, 50 μm.

In addition to the phenotypes described above, *sem-2[Y160C]* mutants also exhibit additional phenotypes, albeit at a much lower penetrance. Instead of having the smooth and tapered tail of wild-type hermaphrodites, 5.4% of homozygous *sem-2(jj471[Y160C])* (*N* = 93) and 7.2% of *sem-2(jj473[Y160C])* (*N* = 111) mutant hermaphrodite L4-adults have an abnormal tail (Fig. 4a–c). This abnormal tail phenotype has not been observed in other *sem-2* mutants, including *sem-2(jj321[P158S])* mutants (*N* > 100, this study) (Tian et al. 2011; Baccas et al. 2025). In addition to the abnormal tail phenotype, 33.3% of *sem-2[Y160C]* mutants (*N* = 30) exhibit a mild constipation phenotype, as evidenced by a slightly expanded intestinal lumen, which is a hallmark of the constipated phenotype (Thomas 1990), compared with WT animals (Fig. 4d and e). This mild constipation phenotype was also observed in *sem-2[P158S]* mutants (Fig. 4f), although at a much lower penetrance (6.7%, *N* = 30).

### The *sem-2[Y160C]* mutation is a recessive, loss-of-function mutation

The human CSS patient with the *SOX11[Y116C]* mutation is heterozygous in the *SOX11* locus (Tsurusaki et al. 2014a). To determine if this mutation causes a dominant gain-of-function effect, a dominant-negative effect, or haploinsufficiency, we examined the *sem-2[Y160C]/+* heterozygous animals for any of the defects that we described above. We generated strains by balancing the *sem-2(jj471[Y160C])* or *sem-2(jj473[Y160C]*) mutation with the *hT2[qIs48] (I;III)* balancer chromosome that contains *myo-2p::gfp*, such that heterozygous *sem-2(jj471[Y160C])/hT2[qs48]* and *sem-2(jj473[Y160C])/hT2[qs48]* animals have a green pharynx due to their expression of *myo-2p::gfp*, while homozygous *sem-2(jj471[Y160C])* and *sem-2(jj473[Y160C]*) animals do not have a green pharynx. The *hT2* balancer chromosome is a reciprocal translocation between chromosome I and chromosome III, and reciprocal translocations can lead to aneuploid progeny (Edgley et al. 2006).

We found that *sem-2(jj471[Y160C])/hT2[qs48]* and *sem-2(jj473[Y160C]*)/*hT2[qs48]* heterozygous animals have a single vulva, lay eggs normally and have a normal brood size (Table 3; Fig. 2c, g), similar to *sem-2(ok2422[null]/hT2[qs48])* heterozygous animals (Table 3; Fig. 2d, h). In addition, we found no difference in the level of *jjIs3900[hlh-8p::nls::mCherry]* expression in heterozygous *sem-2(jj473[Y160C]*)/*hT2[qs48]* animals compared with *sem-2(ok2422[null])*/*hT2[qs48]* animals or to WT animals (Fig. 3a–b’, d–d’, g). Collectively, these results suggest that the *sem-2(jj473[Y160C]*) mutation does not appear to have a dominant negative effect. Instead, it behaves as a recessive and loss-of-function mutation.

## Discussion

Vertebrates have 3 SoxC proteins, SOX4, SOX11, and So12 (Wegner 1999). In humans, mutations in SOX4 and SOX11 are associated with CSS, which is a developmental disorder (Schrier et al. 2012; Sekiguchi et al. 2019). One of the CSS-associated mutations in SOX11 is a heterozygous mutation Y116C (Tsurusaki et al. 2014a). The patient with this mutation presents with microcephaly, hypoplastic fifth toenails, mild intellectual disability, midface hypoplasia, hypertrichosis, arched eyebrows, low-set ears, auricular back-rotation and full cheeks (Tsurusaki et al. 2014a). In this study, we modeled this mutation in the *C. elegans* SoxC protein, SEM-2. Unlike in humans, a single copy of *sem-2* is sufficient for a general wild-type phenotype (Tian et al. 2011). In *C. elegans*, the equivalent of the CSS-associated *SOX11[Y116C]* mutation, *sem-2[Y160C]*, behaves as a loss-of-function, recessive mutation, which suggests that defects in human patients with the *SOX11[Y116C]* mutation are due to haploinsufficiency.

### Insight into the structure-function relationship of SoxC proteins

Sox proteins are characterized by their N-terminal Sox (SRY-related HMG box) DNA-binding domain. One important difference between HMG box-containing proteins that bind DNA in a sequence-specific manner and those that bind DNA in a non-sequence specific manner is the structure of the C terminus of the HMG box (Murphy et al. 1999; Remenyi et al. 2003). Some highly conserved residues near the C-terminal tail of the HMG box of Sox proteins that bind DNA in a sequence-specific manner are thought to contribute to their sequence-specificity (Remenyi et al. 2003). Additionally, Sox proteins bind to the minor groove in DNA and cause a pronounced kink in the DNA double helix, and this kink conformation appears to depend on the highly conserved amino acids and unique features of the C-terminal tail of the HMG box (Jauch et al. 2012).

Mutations in the highly conserved Sox DNA-binding domain, including the highly conserved C-terminal tail region, in SoxC proteins, are associated with CSS (Bowles et al. 2000; Lefebvre et al. 2007; Hempel et al. 2016; Schock and LaBonne 2020). *SOX11[Y116C]* is one such mutation, and its equivalent in SEM-2 is Y160C. We have previously characterized a mutation that is located close to this residue, *sem-2[P158S]* (Baccas et al. 2025). We showed that *sem-2[P158S]* mutant animals exhibit partial embryonic lethality, are egg-laying defective (Egl) due to defects in postembryonic mesoderm M lineage development, and have a low penetrance of the bivulval phenotype (Baccas et al. 2025). Unsurprisingly, the phenotypes of *sem-2[Y160C]* mutants are similar to those of *sem-2[P158S]* mutants. However, the *sem-2[Y160C]* mutation appears to cause a more severe phenotype than the *sem-2[P158S]* mutation. *sem-2[Y160C]* mutants display 65.7% lethality compared with *sem-2[P158S]* mutants that display 30.3% lethality (Table 3 [Baccas et al. 2025]). The bivulval phenotype is also more penetrant in *sem-2[Y160C]* mutants (39.3%) compared with *sem-2[P158S]* mutants (6.9%) (Table 3; [Baccas et al. 2025]). Additionally, as shown in Fig. 3, there is undetectable expression of *jjIs3900[hlh-8p::nls::mCherry]* in the M cell of *sem-2[Y160C]* mutants compared with *sem-2[P158S]* mutants where *jjIs3900[hlh-8p::nls::mCherry]* expression is significantly reduced but detectable (Fig. 3). Finally, *sem-2[Y160C]* mutants exhibit hermaphrodite tail defects that are not observed in *sem-2[P158S]* mutants, while the mild constipation phenotype is more penetrant in *sem-2[Y160C]* mutants (33.3%) compared with *sem-2[P158S]* mutants (6.7%) (Fig. 4). Taken together, these data suggest that the *sem-2[Y160C]* mutation disrupts SEM-2 function more than the *sem-2[P158S]* mutation. It is possible that Y160 plays a more important role in the structure and function of SEM-2 than P158. Based on structural homology modeling, Y160 is closer than P158 to the DNA-contact residue, Y162, in the C-terminal tail region of SEM-2 (Fig. 1 [Jauch et al. 2012]). Additionally, the tyrosine residue equivalent to SEM-2 Y160 / SOX11 Y116 in DNA-bound SRY/SoxA has been found to be involved in prominent side chain interactions among amino acids in the C-terminal region of the HMG box, and these interactions were not observed in the unbound state of SRY (Weiss 2001). Further, Remenyi et al. (2003) suggest that the equivalent of P158 and Y160 play an important role in the ordering of the C-terminal tail region of Sox proteins.

Studies have also shown that the C-terminal tail region of the HMG box in Sox proteins is essential for protein-protein interactions (Remenyi et al. 2003; Wissmuller et al. 2006). The C-terminal tail region of the SOX2/SoxB1 DNA-binding domain forms a protein-protein interface when in complex with Oct1 on the FGF4 enhancer (Remenyi et al. 2003). Thus, it is possible that the *sem-2[Y160C]/SOX11[Y116C]* mutation affects SEM-2/SOX11 function by disrupting protein-protein interaction, in addition to protein-DNA interaction.

We have previously found that *hlh-8/Twist* is likely a direct target gene of SEM-2 in the *C. elegans* postembryonic mesoderm M lineage (Baccas et al. 2025). There is significantly reduced expression of an *hlh-8/Twist* transcriptional reporter, *jjIs3900[hlh-8p::nls::mCherry],* in *sem-2[Y160C]* mutants. This phenotype is consistent with previous molecular analyses of the *SOX11[Y116C]* mutation on target gene expression (Tsurusaki et al. 2014a). The authors conducted luciferase assays using the GD5 promoter in Hela and ATDC5 cells and found that the SOX11[Y116C] mutant protein had significantly less target gene expression compared with WT (Tsurusaki et al. 2014a). They further showed that this significant loss of target gene expression is unlikely due to the abnormal folding or stability of the mutant SOX11 protein because they could detect the SOX11[Y116C] mutant protein expressed in HeLa or ATDC5 cells, and they argued that the mutation causes a low free energy change making it unlikely to significantly affect the folding of the DNA-binding domain (Tsurusaki et al. 2014a). Although we have not examined the expression, localization or stability of the SEM-2[Y160C] protein, we have previously shown that the *sem-2[P158S]* mutation does not affect the expression, localization, or stability of SEM-2 (Baccas et al. 2025). It is therefore likely that the mutant SEM-2[Y160C]/SOX11[Y116C] protein is defective in protein-DNA or protein-protein interaction, thus compromising its ability to regulate downstream gene expression.

### Regulatory relationships that are possibly conserved and affected in coffin-siris syndrome

SoxC proteins regulate the expression of *Twist* in mammalian disease initiation and progression, particularly in the regulation of epithelial-mesenchymal transition (Tiwari et al. 2013; Huang et al. 2015). We have previously shown that SEM-2/SoxC regulates the expression of *hlh-8/Twist* in *C. elegans* postembryonic mesoderm M lineage development (Baccas et al. 2025). Consistent with this regulatory relationship, mutations in *hlh-8/Twist* and in *sem-2/SoxC* cause a similar Egl phenotype due to defects in M lineage-derived vulval muscles, and both *hlh-8* and *sem-2* mutants exhibit differential expression of HLH-8 target genes, including *arg-1/JAG1* and *egl-15/FGFR* (Corsi et al. 2000; Corsi et al. 2002; Meyers and Corsi 2010; Kim et al. 2017; Gruss et al. 2023; Baccas et al. 2025). In humans, defects in SoxC, Twist1, Twist2, JAG1, Notch2, and FGFRs are associated with developmental abnormalities, including craniofacial defects (Muller et al. 1997; Penton et al. 2012; Schrier et al. 2012). The similarities among the mutant phenotypes of SoxC, Twist, and Twist target genes contribute to the labeling of the Egl phenotype in *C. elegans* as a phenolog of craniofacial defects in humans (Kim et al. 2017; Gruss et al. 2023; Baccas et al. 2025).

Constipation is another phenolog of craniofacial defects in humans (Gruss et al. 2023). *hlh-8/Twist* null mutants display constipation and a tail bump or Dar (deformed anal region) phenotype that are thought to be due to defective enteric muscles (Corsi et al. 2000; Kim et al. 2017). HLH-8 regulates the expression of *arg-1/JAG1* in the enteric muscles (Zhao et al. 2007). In this study, we show that *sem-2[Y160C]* mutants exhibit a low penetrant, mild constipation and tail bump or Dar phenotype (Fig. 4). Thus, SEM-2/SoxC likely functions in the development of the enteric muscles via regulating the expression of *hlh-8/Twist* and hence the expression of HLH-8/Twist target genes, during enteric muscle development.

CSS is most often caused by mutations in components of the BAF, or SWI/SNF, complex, in addition to mutations in SOX4 and SOX11 (Miyake et al. 2014; Tsurusaki et al. 2014b). The relationship between the BAF complex and SOX11 appears complex. SOX11 has been shown to be a key downstream target of the Pax6-BAF complex in driving neurogenesis (Ninkovic et al. 2013). Inversely, SOX11 has been identified as a core transcription factor in adrenergic high-risk neuroblastoma, where SOX11 can regulate the expression of multiple SWI/SNF subunits in adrenergic neuroblastoma cells (Decaesteker et al. 2023). It has been suggested that SOX11 may promote proliferation in the facial mesenchyme in its role in regulating craniofacial development (Schock and LaBonne 2020). Components of the BAF or SWI/SNF complex are conserved in *C. elegans*, and SWI/SNF subunits are known to function in a dose-dependent manner to regulate entry of and exit from the cell cycle during M lineage development (Ruijtenberg and van den Heuvel 2015; van der Vaart et al. 2020). In particular, partial depletion of SWI/SNF components, such as SWSN-1/SMARCC1/SMARCC2, SWSN-4/SMARCA2/SMARCA4, and SWSN-8/ARID1, results in an over-proliferative phenotype in the sex myoblast (SM) lineage, a subset of the M lineage, while complete removal of SWI/SNF subunits leads to cell division arrest in the SM (sex myoblast) lineage (van der Vaart et al. 2020). M lineage cell proliferation is also sensitive to changes in functions of SEM-2/SoxC and HLH-8/Twist (Corsi et al. 2000; Corsi et al. 2002; Tian et al. 2011; Baccas et al. 2025). Forced expression of *sem-2* in the M lineage results in the transformation of M-derived body wall muscles and coelomocytes, which represent terminally differentiated fates, to the proliferative SM fate, while *sem-2* mutants with no *sem-2* expression in the SM lineage have an SM to body wall muscle fate transformation (Tian et al. 2011). In addition, *sem-2[P158S]* mutants have an SM proliferation defect (Baccas et al. 2025). Similarly, animals with a semidominant allele of *hlh-8/Twist, n2170[E29K],* have SMs that often fail to divide (Corsi et al. 2002). Given the involvement of SEM-2/SoxC and SWI/SNF in cell proliferation vs cell differentiation during M lineage development, it is possible that SEM-2 and subunits of the SWI/SNF complex exhibit regulatory relationships. Future studies will be needed to decipher these relationships.

In vitro systems are useful for initially investigating the molecular effects of disease-associated mutations, but the use of in vivo models is crucial for accurate assessment given the dynamic nature of proteins and their sensitivity to the intra- and extra- cellular environment, cell signaling, and protein–protein interactions. In recent years, model organisms including *C. elegans* have contributed to the discovery, as well as mechanistic understanding of rare undiagnosed diseases (Amsalem et al. 2016; Kim et al. 2017; Boulin et al. 2021; Fielder et al. 2022; Gruss et al. 2023). Our data provide further support of the use of *C. elegans* to model CSS and other genetic craniofacial abnormalities, in particular, due to established phenologs of craniofacial defects, which are useful for placing key regulatory proteins associated with craniofacial development in gene regulatory networks and for dissecting their structure-function relationships.

## Data Availability

The authors affirm that all data used to make conclusions are within the article.
